# Automated Analysis of Pollutants in Wastewater Using Atmospheric Pressure Gas Chromatography‐Tandem Quadrupole Mass Spectrometer With Robotic Autosampler and Solid Phase Micro Extraction

**DOI:** 10.1002/jssc.70328

**Published:** 2025-11-25

**Authors:** Dnyaneshwar Shinde, Urvikkumar Dhagat, Vijayakumar Murugan, Parth Gupta, Raghu Tadala, Bhaskar Karubothula, Chaitanya Devireddy, Edward Stanislaus, Samara Bin Salem, Wael Elamin, Grzegorz Brudecki

**Affiliations:** ^1^ RASID Laboratory of Abu Dhabi Quality & Conformity Council (ADQCC) & M42 Environmental Sciences Abu Dhabi United Arab Emirates; ^2^ Abu Dhabi Quality and Conformity Council UAE Abu Dhabi United Arab Emirates

**Keywords:** automated extraction, chemical contaminants, Green Chemistry, verification

## Abstract

The analysis of organic pollutants using gas chromatography coupled to mass spectrometer (GC‐MS) with electron impact (EI) ionization and liquid–liquid or solid‐phase extraction (LLE or SPE) often suffer from a limited selectivity and arduous sample preparation. Therefore, atmospheric pressure GC‐MS/MS coupled with Dual Head‐Robotic Tool Change (DH‐RTC) Prep and Load (PAL), and solid‐phase micro extraction (SPME) arrow was investigated for its suitability. Evaluation of the SPME arrow coated with divinylbenzene–polydimethylsiloxane (DVB‐PDMS) using 2‐nitrotoluene and nitrobenzene showed acceptable intra‐day precision (%relative standard deviation [RSD]) of less than 10% and inter‐day precision (*n* = 35) of 10.54% and 12.45% respectively. Initial trials were conducted to assess the suitability of technique for eight multiclass compounds (from early eluting volatile organic compounds [e.g., 1,2‐dichlorobenzene] to challenging late eluting polycyclic aromatic hydrocarbons [PAH] [e.g., benzo[ghi]perylene], specification markers [e.g., octafluoronaphthalene, and hexachlorobenzene], labile and toxic analytes [e.g., endosulfan, and 2,3,7,8‐tetrachlorodibenzodioxin], and isobaric closely eluting PAHs [phenanthrene, and anthracene]). The precision (%RSD, *n* = 6) was ranged from 1.68 to 16.57, with peak asymmetry factor between 1.003 (phenanthrene) and 1.520 (anthracene). Further experiments conducted for priority pollutants (United States Environmental Protection Agency (U.S. EPA) 625.1/2016) indicated that the addition of salt significantly enhanced the responses for 28 analytes. The increase in response was ranged from 1.14‐fold for Endosulfan II to 40.53‐fold for pentachlorophenol. However, the %RSDs for 51 analytes were better without adding salt and for 12 analytes it improved with the addition of salt. Since the responses for 12 analytes are sufficient even without salt and standard deviations are below U.S.EPA 625.1/2016 limits, further trials were conducted without adding salt. Preliminary trials showed high RSDs up to 52.76% for high‐boiling compounds, which were reduced by using internal standards (IS) and lowering the analyte load during injection. Dedicated analysis of high‐boiling PAHs and selected compounds with deuterated IS achieved acceptable coefficient of determination (*r* ^2^), %recoveries and %RSDs. The evaluation of method greenness (Green Analytical Procedure Index and Analytical Greenness calculator), and practicality (Blue Applicability Grade Index) indicated that this approach is environmentally sustainable and practical, providing an efficient and validated alternative method for wastewater analysis.

## 1 Introduction

Semi‐volatile organic pollutants such as organo‐chlorine pesticides (OCPs), polycyclic aromatic hydrocarbons (PAHs), phthalates, phenols, and other semi‐volatile organic compounds (SVOCs) are conventionally analyzed by liquid–liquid extraction (LLE) or solid‐phase extraction (SPE). Detection is carried out using electron impact (EI) ionization coupled with gas chromatography‐mass spectrometry (GC‐MS) or liquid chromatography with specific detectors such as electron capture, flame ionization, ultraviolet, and fluorescence detector [1, 2, 3, 4, 5, 6, 7, 8, 9, 10, 11, 12, 13]. These traditional workflows require significant manual efforts and the use of large quantities of solvents such as dichloromethane (DCM), followed with the analytical steps like large volume extractions, filtration, clean‐up (gel permeation chromatography [GPC] and column chromatography), concentration, evaporation, and reconstitution. Because of these complex steps involved, conventional methods often face issues like low throughput and poor environmental sustainability. The use of DCM presents environmental and regulatory concerns, with the US EPA finalizing a ban on most uses (April 30, 2024), due to its hazardous nature [14]. Sample preparation is one of the most limiting factors of these conventional methods [15]. Therefore, there is a need of alternative approaches that minimize or eliminate carcinogenic solvents, automate analytical steps for high throughput, and improve sensitivity and accuracy.

Solid‐phase micro extraction (SPME) is a solvent free sample preparation technique that directly extracts suitable analytes from a sample with an extraction phase coated on a small diameter fused silica fiber [16]. The phase coated fiber can be used for direct immersion (DI‐SPME) in a liquid sample or headspace (HS‐SPME) sampling, where fibers are exposed to liquid sample or headspace above the thermally equilibrated liquid sample vial. Over the past few decades, SPME fibers has become a commonly used extraction technology for environmental, food, and clinical analysis. However, this technology has faced certain limitations due to the initial design of the SPME fibers, such as limited stability, small phase volumes with limited durability, and the necessity for manual intervention during sample extraction and injection [17]. Most common challenges of traditional SPME fibers are a lack of physical durability of the fused silica, fragility due to the delicate mechanical setup and most of the fibers cannot be repaired once damaged [18].

After the expiration of intellectual property rights (IPR), research groups came up with mechanically and chemically enhance versions of this technology. An example of these enhancements includes the first large volume SPME fibers developed by CTC Analytics AG for GC applications, commonly known as the SPME Arrow [19]. SPME Arrows were developed to address the shortcomings inherent in the workflow of conventional SPME fibers. SPME Arrow has superior physical attributes, including a larger septum piercing needle diameter (1.1 and 1.5 mm) compared to traditional SPME fibers (0.573 and 0.511 mm), making it about 2–3 times thicker [3]. These increased external diameters are largely responsible for the increased mechanical robustness of SPME Arrows [19]. The SPME Arrows are named for their “arrow” shaped tips, which have same external diameter as that of SPME Arrows' septum piercing needle (i.e., 1.1 and 1.5 mm). The Arrow tip enhances the mechanical robustness of the SPME Arrow. SPME Arrow requires less force (799 g for 1.1 mm diameter SPME Arrow and 908 g for 1.5 mm diameter SPME Arrow) to penetrate the vial or GC inlet septa compared to a traditional SPME fibers (1188 g for 0.63 mm diameter SPME fibers), even with the increased diameter [20]. The SPME Arrow tip cuts slits in vial and GC inlet septa instead of coring them. As a result, septa last as long as or even longer than with traditional SPME fibers [21]. In addition, when retracted, the Arrow tip acts as a protective cap, reducing the diffusion of compounds into the septum piercing needle and reaching the phase. This reduces background contamination and prevents analyte loss while storing fibers for GC system [22].

**FIGURE 1:**
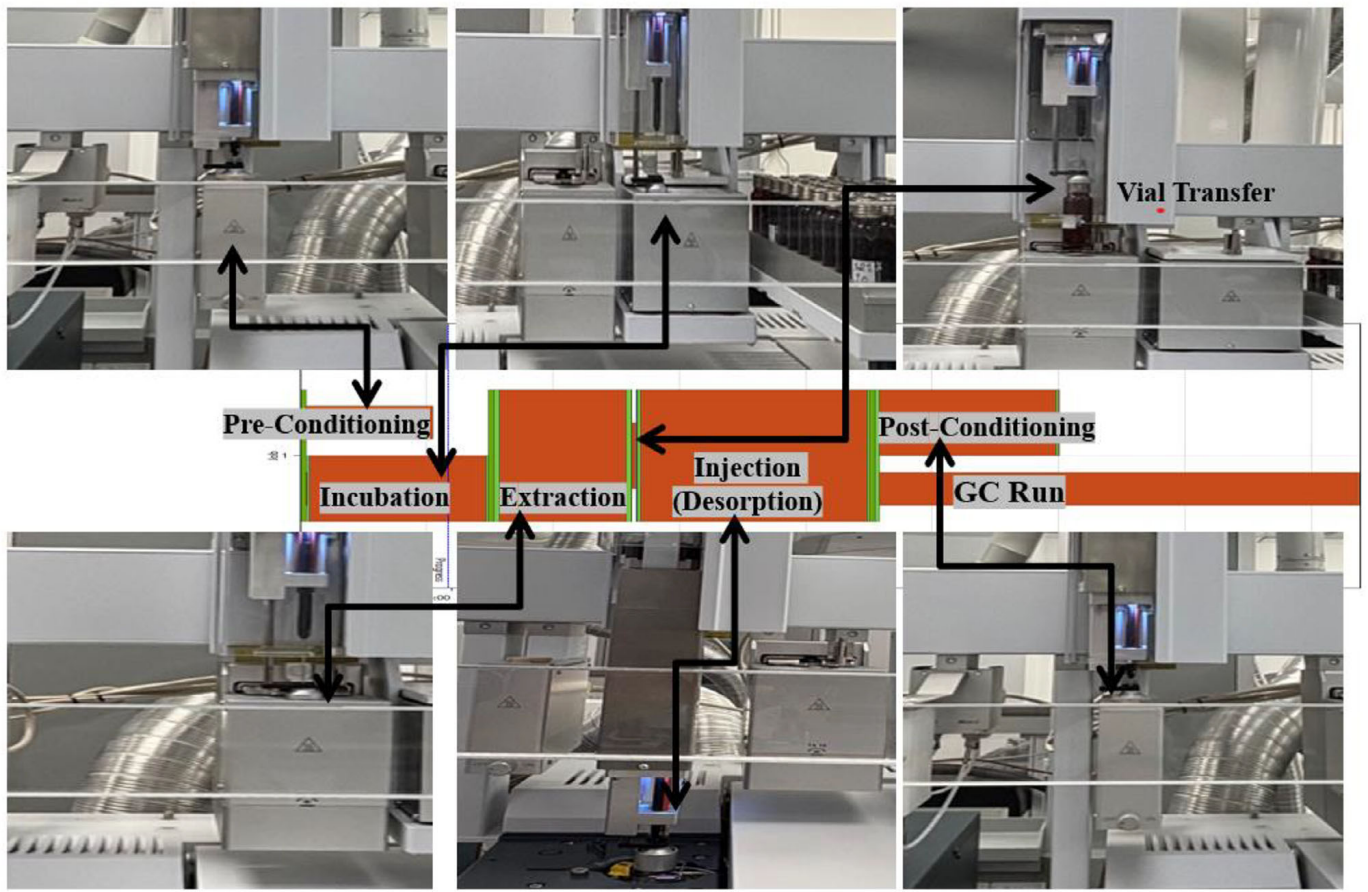
APGC‐MS/MS with DH‐RTC PAL autosampler for SPME arrow extraction.

Further, manual extractions and desorption appear to increase traditional SPME fibers failure rates. With the advancements in instrumentation, SPME Arrow‐based analysis evolved with time by combining sample preparation and sample introduction systems on one device. The modular autosampler Dual Head Robotic Tool Change (DH‐RTC) Prep and Load (PAL) (CTC Analytics AG, Zwingen, Switzerland) is equipped with tools for liquid injection, SPME arrow extraction tool using a heated agitator and stirrer, a vortex, a fiber conditioning station and washing station has the capacities to automate the sample preparation and sample introduction on APGC‐MS/MS (Figure 1) without manual intervention.

In GC–MS/MS, EI ionization is commonly used for its capability to ionize a wide range of organic compounds. It is widely recognized and accepted that the EI source results in extensive fragmentation of molecules, which leads to the absence of molecular ions in most cases of EI spectra [1]. The lack of quasi‐molecular ions in MS spectra affects sensitivity and specificity of the analysis. Chemical ionization (CI) or high‐resolution mass spectrometry (HRMS) can offer additional details for analysis [23, 24]. It has been observed in the literature that, in addition to the selectivity advantage arising from the ability to use the quasi‐molecular ion, sensitivity is also substantially improved with the use of APGC‐MS/MS [25].

The objective of the current study is to evaluate an automated and high‐throughput alternative procedure to the complex conventional methods for the analysis of multiclass semi volatile organic pollutants in water matrices. This study assessed the suitability of DH‐RTC PAL with SPME Arrow connected to an APGC system coupled with a tandem quadrupole (QqQ) mass spectrometer. The proposed method underwent performance verification of SPME Arrows and evaluation of the technique's suitability using a test mix of eight GC‐compatible multiclass challenging compounds. Followed by assessment of the impact of salt addition on precision and response for priority compounds listed as per USEPA 625.1/2016 [1] with comparative evaluation of method performance. Finally, the method was assessed for its quantitation capabilities using internal standard (IS) and strategic selection of number and concentration of the analytes. Furthermore, the method was assessed for its eco‐friendliness and practicality using tools like the Complex Modified Green Analytical Procedure Index (ComplexMoGAPI), Analytical Greenness (AGREE), and Blue Applicability Grade Index (BAGI). To the best of the authors' knowledge, this is the first comprehensive study to develop, evaluate, and comparatively assess a fully automated method for the analysis of 70 multiclass semi volatile organic pollutants while also demonstrating its environmental sustainability and practical applicability through a greenness assessment.

## 2 Experimental

### 2.1 Chemicals and Materials

The Certified Reference Materials (CRMs) of 70 pollutants and 49 Isotopically Labelled Internal Standards (ILIS) were purchased from Dr. Ehrenstorfer (Augsburg, Germany). Stock standard solutions (around 1000 mg/mL) were prepared by dissolving the reference standards in acetonitrile and stored in a freeze at 4°C. Intermediate standard mixtures were prepared by volumetric dilution of stock solutions (10 mg/mL) in ethyl acetate. Working standard mixtures were prepared by volume dilution of the intermediate solutions (1 mg/mL) in ethyl acetate. SPME Performance Test Mix, 1 µg/mL, in water/methanol (99:1) from Restek (Catalogue No. 31015) was used for evaluation of intra and inter day precision. A System Suitability Test Mix (SSTM) for APGC‐MS/MS from Waters (Catalogue No. 700005254‐7) was used for the evaluation of suitability of this novel instrument configuration for multiclass compounds.

Ethyl acetate, acetonitrile and anhydrous sodium sulfate were purchased from Honeywell Speciality Chemicals (Seelze, Germany), Scharlab S.L. (Sentmenat, Spain) and Fisher Scientific (Geel, Belgium) respectively. Solvents used were of MS grade, and the sodium sulfate used met purity standards set by American Chemical Society (ACS) and was anhydrous with purity of more than 99%. SPME Arrows featuring a DVB/PDMS phase (120 µm × 20 × 1.1 mm) and 20 mL amber vials with caps were procured from CTC Analytics AG, Zwingen, Switzerland. Wastewater control samples used for method optimization and verification were prepared by collecting and mixing 100 pre‐screened control samples from different domestic and industrial sites in Abu Dhabi and Al Ain cities of United Arab Emirates. This representative control wastewater sample was used in study trials to plot procedural calibration curve and quality control checks for the method verification. ASTM Type I water used in method optimization and verification was obtained from a Merck ultrapure water purification system.

### 2.2 Instrumentation for SPME Arrow Extraction, Mass Spectrometric and Gas Chromatographic Method

The data was acquired using a GC system (Agilent 8890B, Palo Alto, CA, USA) coupled to a tandem quadrupole (QqQ) mass spectrometer (Xevo TQ‐XS, Waters Corporation, Manchester, UK), with the source operating in atmospheric pressure (that is APGC mode) and equipped with DH‐RTC PAL (CTC Analytics AG, Zwingen, Switzerland). Chronos software (version 4.16.28330.0) was used to control, create, and load batch sequences using the DH‐RTC PAL autosampler. MassLynx (version 4.2) software was used for data analysis.

The DH‐RTC PAL autosampler equipped with holding tools, parking stations, a tray holder, SPME Arrow conditioning module, vortex, an agitator, and stirrer for SPME Arrow extraction as shown in Figure 1 was used for the extraction of the analytes. The extraction procedure started by transferring 20 mL of the capped sample vial, containing 5 mL of the sample, onto an agitator, where sample was incubated for 5 min to reach thermal equilibrium. Meanwhile in parallel, SPME Arrow was subjected to pre‐conditioning for 5 min at 260°C under N_2_ stream. After simultaneous sample incubation and pre‐conditioning of the SPME Arrow the sample vial was transferred for extraction in a stirrer using robotic arm. The extraction of the sample was done by exposing the SPME arrow phase to the headspace of thermally equilibrated vial for optimized time and temperature combination. The adsorbed or absorbed analytes on SPME Arrow phase were injected on APGC injector port by desorption at 280°C for 9 min. Thereafter the SPME arrow was subjected to a post conditioning after desorption to ensure a clean phase for the next sample. A fused silica Select PAH GC capillary column (length 30 m × I.D. 0.25 mm × film 0.15 µm) (Agilent part no. CP7462 J&W Scientific, Folsom, CA, USA) was used for APGC separation. A deactivated capillary (column without stationary phase) guard column (0.75 m) and retention gap (0.75 m) were connected to the front and back end of the main column, using multi union kit. Details of the optimized automated extraction, gas chromatographic and mass spectrometric method parameters are given in Table 1.

**TABLE 1:** Analytical conditions for automated extraction and Detection of SVOCs in wastewater.

Sr. No.	Automated extraction		APGC method parameters		MS method parameters	
	DH‐RTC PAL with SPME arrow		Agilent GC 8890		Waters TQ‐XS (APCI source)	
1	Sample	5 mL	Inlet type	MMI‐SSL	Corona current	2 µA
2	SPME Arrow	DVB/PDMS (120 µm × 20 × 1.1 mm)	Liner	Spitless 4.0 mm id	Auxiliary gas flow (N_2_)	200 L/h
3	Pre‐conditioning	5 min at 260°C under N_2_ stream	injection mode	Spitless	Cone (C) gas flow (N_2_)	260 L/h
4	Incubation	5 min at 80°C, stirring at 500 rpm	Injector temp.	280°C	Collision gas flow (Ar)	0.15 mL/min
5	Extraction (HS)	5 min at 80°C, stirring at 250 rpm	Septum purge	3 mL/min	Source temp.	150°C
6	Injection (Desorption)	APGC inlet at 280°C for 9 min.	APGC column	Select PAH, (30 m × 0.25 mm × 0 .15µm)	MS method events	Corona 20 µA (first 3 min), then 2 µA. Cone Gas 50 L/h (first 3 min), then 260 L/h
			Carrier gas	He @ 2 mL/min		
			Transfer line temp	330°C		
7	Post‐conditioning	5 min at 260°C under N_2_ stream	APGC oven program	50°C hold for 1 min, 120°C @ 10°C/min, 320°C @ 20°C/min hold 10 min.	acquisition mode	MRM

The mass spectrometric parameters were finely tuned using individual CRMs along with IS. The tuning solutions of individual CRMs were prepared at 1000 ng/mL in ethyl acetate. Mass spectrometric method parameters for quantifier and qualifier ions including cone voltage (CV), and collision energy (CE) were optimized by injecting 1 µL of tuning solution. During initial multiple reaction monitoring (MRM) method optimization, the DH‐RTC PAL with liquid injection tool was used for injection of 1 µL using 10 µL syringe with injection and fill speeds of 100 and 20 µL/s respectively. The injection was performed with six pre‐cleaning cycles of ethyl acetate and six post‐cleaning cycles of acetonitrile to avoid carryover. Quantifier (Q1) and Qualifier (Q2) ions were accurately selected for all analytes to get better selectivity and sensitivity. Segmented MRM method was created based on the retention times of analytes to get a minimum of 12 points per peak, for all MRM channels as shown in Table S2.

### 2.3 Data Analysis, Calculation, and Representations

Data analysis was performed using TargetLynx (version 4.1.1.0) module of MassLynx (version 4.2), and the Microsoft Office 365 Excel worksheet was used for calculation of accuracy, repeatability (as %relative standard deviation [RSD]) and plotting charts for data representation. The results of the effect of salt addition trials were statistically analyzed using Statistical Package for the Social Sciences (SPSS 30.0) software (IBM SPSS Statistics Version 30.0.0.0 (172), 2024, Armonk, NY) for one‐way analysis of variance (ANOVA). Online tools were used for evaluation of method greenness and its applicability (Table S2).

### 2.4 Evaluation of the Method Greenness and Its Applicability

With the increasing focus on sustainable environments and green chemistry principles, several tools have been used to evaluate the greenness and practicality of various analytical methods. The proposed method was evaluated for its eco‐friendliness using two tools: the Green Analytical Procedure Index (GAPI) and the AGREE analytical greenness (AGREE). In addition, its practicality for high‐throughput analysis was evaluated using the BAGI. GAPI is a semi‐quantitative tool designed to assess the environmental impact of analytical methods. It evaluates analytical steps like sampling, sample preparation, reagents, energy use, and other factors, using a color‐coded pentagram (green, yellow, and red) to show environmental impact. The latest version, ComplexMoGAPI, was utilized to assess method greenness alongside the AGREE analytical greenness calculator [26]. AGREE evaluates the method on 12 principles of Green Analytical Chemistry (GAC), which are sampling procedure, sample volume, positioning of analytical device, number of sample preparation steps, sample preparation technique, derivatization, amount of waste generation, number of analytes in single run, instrument type, source of reagent, reagent toxicity, and operators' safety [27]. The BAGI is used to assess the practicality of analytical methods, assigning scores between 25 and 100, with higher scores indicating greater practicality [28].

## 3 Results and Discussion

### 3.1 Outcome of Method Optimization

During the optimization of mass spectrometric method, 51 out of the 70 targeted analytes exhibited quasi molecular ions or protonated ions (M+H), or characteristic chlorine isotopic (^35^Cl or ^37^Cl) molecular ions in the mass spectra of GC‐MS/MS with source at atmospheric pressure and soft ionization (Table S2). In the USEPA 625/2016 method, the molecular ion is used as the primary ion with an EI source for 34 of the 66 priority molecules. For an additional five molecules, CI using methane was specified to generate the quasi‐molecular ion as the primary ion [2]. The comparative evaluation with U.S. EPA 625/2016 for primary ion selection revealed that molecular ions were abundant in the APGC‐MS/MS mass spectra for 13 additional compounds: endosulfan sulfate, endrin, 4,4′‐DDE, Di‐n‐octyl phthalate, bis(2‐Ethylhexyl) phthalate, Butyl benzyl phthalate, Di‐n‐butyl phthalate, Diethyl phthalate, 2,4‐Dinitrotoluene, 2,6‐Dinitrotoluene, Isophorone, Nitrobenzene, and 4‐Nitrophenol (Table S2). In case of 14 OCPs, one phthalate (Dimethyl Phthalates) and three other SVOCs (bis 2 chloroisopropyl ether, hexachloroethane, and bis 2 chloroethoxy methane), the second largest precursor ion with maximum abundance in the mass spectra was selected. The significant presence of quasi‐molecular ions for 5 phthalates was observed, whereas other studies mostly use *m*/*z* 149 as a base peak ion for phthalates due to in‐source fragmentation [29, 30, 31]. Using *m*/*z* 149 as a base peak for the analysis of phthalates by mass spectrometry presents several analytical challenges. It is a common fragment ion from matrix interferences, plasticizers, glassware, dust, plastic laboratory consumables, and stationary phase of GC columns and septa bleed. The ubiquitous presence of phthalate esters can result in overestimation, false positive identification, and incorrect identification of individual phthalates [31]. The presence of quasi molecular ions in most APGC‐MS/MS spectra and larger base peak ions for compounds with in‐source fragmentation demonstrate better selectivity compared to conventional methods for testing semi‐volatile organic pollutants (Table S3).

During gas chromatographic method optimization, a notable observation was the change in the elution order of beta and gamma HCH when using a Select PAH (30 m × 0.25 mm × 0.15 µm) column instead of a DB 5MS (30 m × 0.25 mm × 0.25 µm) column. Conventional analyses of OCPs typically use a DB 5MS column, where HCH isomers elute in the order alpha, beta, gamma, and delta [2]. However, in this study, using a more PAH‐selective column with reduced film thickness, the gamma isomer eluted before the beta isomer. The elution order of various structural isomeric compounds remains unchanged when using the novel Select PAH column compared to the DB 5MS column. These compounds include: phenanthrene and anthracene (178), fluoranthene and pyrene (202), benzo(a)anthracene and chrysene (228), benzo(b)fluoranthene, benzo(k)fluoranthene, and benzo(a)pyrene (252), indeno(1,2,3‐cd)pyrene and benzo(ghi)perylene (276), chlordane *cis* and chlordane *trans* (406), alpha‐endosulfan and beta‐endosulfan (404), dieldrin, endrin, and endrin aldehyde (378), bis 2‐ethylhexyl phthalate and Di‐n‐octyl phthalate (390), 2‐nitrophenol and 4‐nitrophenol (139), and 2,6‐dinitrotoluene and 2,4‐dinitrotoluene (182). These pairs exhibit the same molecular mass; however, they are well separated (Table S2) chromatographically. The most challenging pair of the isomer that is benzo (b) and (k) fluoranthene was well separated chromatographically (base line valley < 25%), however it was observed to be difficult to maintain the separation with continuous operation for more than 2 weeks without maintenance. This can be because of the high boiling point (more than 480°C) of these isomers which is higher than the maximum inlet temperature of the APGC, causing incomplete evaporation and deposition of high boiling compounds on the inlet and front end of the APGC column. Therefore, the approach of reporting the results as sum of benzo b and benzo k fluoranthene was considered. If a single isomer is present in the unknown sample, it is recommended to reanalyze with individual peak integration.

Optimization of automated extraction using DH‐RTC PAL with SPME Arrow involve pragmatic evaluation of impact of many physico‐chemical parameters. These parameters include selection of SPME Arrow (phase and thickness), analytes (concentration and compatibility), sample (volume, pH, matrix effect, ionic strength), penetration depths (vial and injector), speed (agitation and stirring), time‐temperature (arrow conditioning, sample incubation, extraction and desorption), injection mode (split/split less), liner (split/split less/ID‐1.7 mm/4 mm), and selection of IS. It is essential to evaluate these parameters individually for each compound or for the entire class. However, this process is time‐consuming and may result in multiple methods required for different classes of compounds, thereby reducing the practicality of the method at the scale of BAGI. Therefore, the goal of this initial study was not to find perfect value of each parameter for every analyte. Instead, it aimed to assess literature values, verify the impact of several parameters on a small scale, and develop an approach which is comparatively suitable for qualitative and/or quantitative analysis of all targeted compounds in one injection. According to preliminary studies, the parameters listed in Table 1 were evaluated for their suitability for automated SPME Arrow extraction in compliance with USEPA625.1/2016 [1].

**FIGURE 2:**
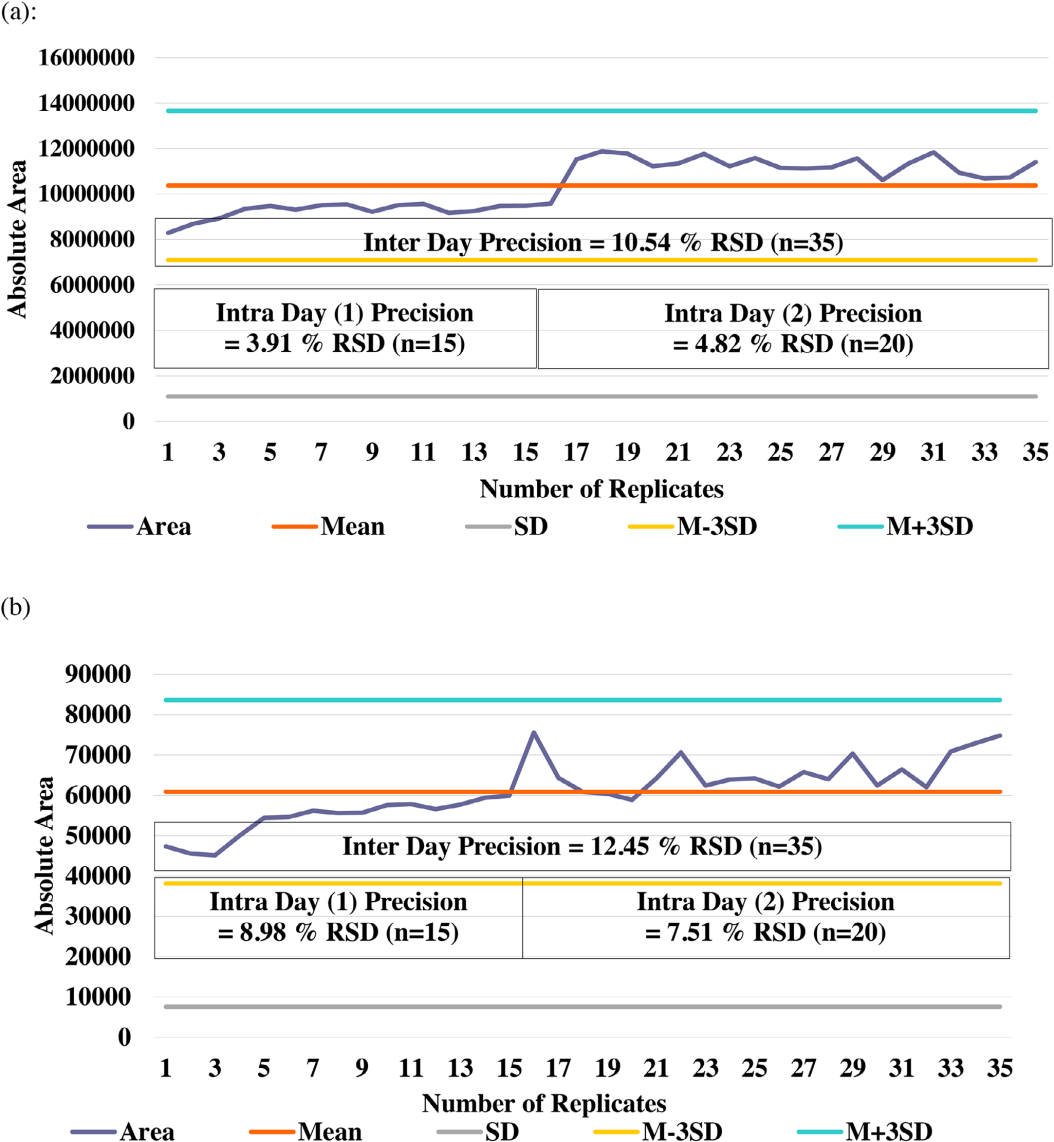
SPME Arrow Performance Test using DH‐RTC PAL with APGC‐MS/MS for precision (a): 2‐nitrotoluene and (b): nitrobenzene.

For SPME Arrow based automated extraction as mentioned in SANTE/11312/2021v2 2024 at point number G4, precision (%RSD) must be demonstrated during verification of method performance [32]. Therefore, parameters in Table 1 were first evaluated for inter‐day (*n* = 15 [Day 1] and *n* = 20 [Day 2]) and intra‐day (*n* = 35) precision of chromatographic peak area using SPME performance test mix, (Restek catalogue No. 31015) containing nitrobenzene and 2‐nitrotoluene at 5 ng/mL. It was observed from the data in Figure 2a, that inter‐day precision in terms of %RSD for 2‐nitrotoluene was 3.91% and 4.82% with the intra‐day precision of 10.54%. Similarly in case of nitrobenzene inter‐day precision was 8.98% and 7.51% with the intra‐day precision of 12.45% RSD (Figure 2b). It was observed that the parameters selected from the preliminary trials were suitable for further investigation of method suitability for representative compounds from different class.

### 3.2 Method Verification

The evaluation of the method's applicability was initiated by assessing its suitability for eight representative compounds from various chemical classes in ASTM Type I water. The selected compounds reflect both the technical complexity, regulatory requirement of various analytes and their relevance in assessing the operational health of the APGC‐MS/MS system. These compounds serve as robust performance indicators for validating the method for compounds across a wide range of chemical properties. Selected compounds include: hexachlorobenzene (HCB) and octafluoronaphthalene (OFN) as they are historically used as a reference compound for sensitivity and other GC specifications (Figure S3). Phenanthrene and anthracene are isobaric PAHs that form a chromatographic doublet, showing both charge transfer and protonated ions; useful for assessing chromatographic resolution. Endosulfan it is a labile organochlorine pesticide prone to degradation, highlighting the need for optimal injector and ion source conditions. The most toxic 2,3,7,8 tetrachlorodibenzo p dioxin (TCDD) is a very well‐known dioxin and persistent organic pollutant (POP). Benzo[ghi]perylene is a high‐boiling PAH with tailing issues often shows signal loss over prolonged instrument use without maintenance. 1, 2 Dichlorobenzene is an early‐eluting, low‐boiling compound indicates suitability of the method for volatile organic compounds (VOCs). Again, for these eight representative compounds as mentioned in SANTE/11312/2021v2 2024 at point number G4, area precision (%RSD) along with the peak symmetry factor was evaluated at 0.1 ng/mL (*n* = 6). It was observed from the data in Figure S3 that area precision (%RSD) was ranged from a minimum of 1.68% RSD (for HCB) to a maximum of 16.57% RSD (for Benzo[ghi]perylene). A maximum %RSD of 16.57 was observed for Benzo[ghi]perylene, which can be attributed to its high boiling point (above 550°C) limiting its equilibration and evaporation. However, this area precision observed is less than generally acceptable value of less than 20% RSD. Peak asymmetry factor was evaluated for assessing unwanted interaction of analytes on arrow phase, overloading of the column due to unknown rate of concentration of analytes on arrow phase and incomplete elution due to ad/absorption of analytes on arrow phase. Peak asymmetry factor was observed to be ranged from a minimum of 1.003 (for phenanthrene) to a maximum of 1.520 (for anthracene) [33].

During the second stage of method verification, the applicability of the method was assessed for a targeted list of 66 priority pollutants as specified in the U.S. EPA 625:2016 method. Salt addition plays an important role during extraction mostly by modifying ionic strength and solubility depending on the chemistry of targeted pollutant. Therefore, during this study area precision was evaluated for pollutants with and without salt addition. It was observed from the data in Table S3 that 63 pollutants remained detectable (*n* = 6) with and without addition of the salt at 2 ng/mL. Further, it was observed that salt addition has helped extraction of 28 compounds as the chromatographic peak area was increased by more than 1‐fold. During the initial trial, inadequate results were observed for three analytes: 2‐methyl‐4,6‐dinitrophenol, benzidine, and 3,3′‐dichlorobenzidine. This can be due to the problematic nature of these compounds, which is evident from the high values of method detection limits (MDLs), minimum levels (MLs), and other method acceptance criteria outlined in EPA625.1/2016. For these compounds, results after properly optimizing the method without adding salt were presented in Table S3.

It was observed that salt addition helped extraction of 10 targeted phenols, as area increase was observed to be from a minimum of 7.44 folds (for 2 4 dichlorophenol) to a maximum of 40.53 folds (for pentachlorophenol). Further, it was evident from the data in Table S3 that salt addition has helped to increase extraction efficiency for compound which are relatively small molecules and/or polar in nature. This can be because of salt usually increase uptake of more polar compounds that have a higher solubility in water. This can be due to increased ionic strength of aqueous phase, decreasing the analytes solubility which promotes their partitioning between the available phases [34, 35]. The data in Table S3 indicates that the addition of salt has negatively impacted the area response and %RSD of all 17 targeted PAHs. However, the results obtained without adding salt are acceptable. Also, it was evident from the data in Table S3 that addition of salt has negatively affected extraction of compounds which are relatively larger molecules and/or non‐polar in nature. This can be because of PAHs, non‐polar and large molecules are inherently hydrophobic, and they have low water solubility, meaning they tend to partition without salting out effect. Moreover, the addition of salt can affect the natural partitioning of these molecules. Various factors such as potential ion pairing or competing salt migration can cause increased %RSDs and decrease the area response. An increase in the response was observed for 28 compounds with the addition of salt; however, %RSDs were better for 51 analytes without the addition of salt. While only 12 analytes showed improved %RSDs with salt addition. The response for these 12 analytes remains adequate without the addition of salt, with a standard deviation lower than that observed in the initial demonstration of capability injections as per USEPA 625.1/2026 Method, (DOC‐Section 8.2). Therefore, it is recommended to proceed without the addition of salt and analyze all analytes in a single run.

**FIGURE 3:**
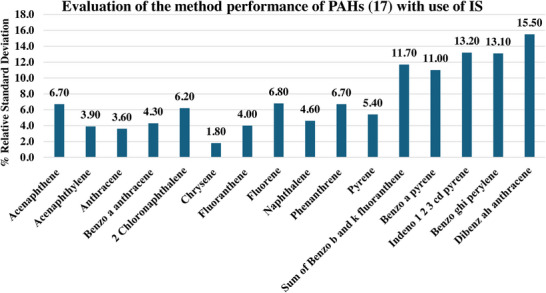
Evaluation of method performance for PAHs (17) using ILIS.

Further, it was observed from the data in Table S3 that %RSDs of specifically high boiling and late eluting compounds (Benzo(ghi)perylene, Dibenz(a,h)anthracene, Indeno (1,2,3‐cd) pyrene, Di‐n‐octyl phthalate, Endrin aldehyde, 4,4′‐DDT, 4,4′‐DDE, Aldrin) was higher (> 35%) with and without addition of salt. The high concentration of analytes, increased number of analytes, and interfering matrix components in wastewater compete for equilibrium in the vial headspace and adsorption/absorption on the SPME Arrow coating, potentially leading to saturation of the extraction phase. The low %RSD of high boiling and late eluting compound (Benzo(ghi)perylene‐16.57) observed during the method applicability study with a smaller number of analytes (eight multiclass compounds) at low concentration (0.1 ng/mL) in interference free matrix (ASTM Type 1 water) supports this conclusion. However, the results meet the QC acceptance criteria in EPA625.1/2016 for “s” that is standard deviation of DOC injections (initial demonstration of capability discussed at Section 8.2) for all compounds without addition of salt. This issue of higher %RSD can be resolved by using ILIS and reducing the number and concentration of analytes during confirmatory analysis. To evaluate and demonstrate the effectiveness, trials were conducted using IS and reducing the analyte load during injection to assess the %RSD of the highest and late eluting PAHs at 1 ng/mL. It was observed from the data in Figure 3 that dedicated analysis of only PAHs with deuterated IS achieved acceptable %RSDs of calculated concentration, ranging from a minimum of 1.80 (for chrysene) to a maximum of 15.50 (dibenz[ah]anthracene).

Further, to assess the potential of SPME Arrow‐based automated extraction and detection by APGC‐MS/MS for quantitative analysis, trials were conducted on a limited number (20) of representative compounds from various chemical classes (Table 2). Compound from the class of VOCs, phenols. Phthalates, PAHs, OCPs, POPs, and other SVOCs were selected and quantified using five‐point calibration curve with IS and accuracy (% Recovery ± %RSD) was evaluated at 1 ng/mL (*n* = 6). It was observed from the data in Table 2 that coefficient of determination (*r* ^2^) of procedural calibration curve was ranged from a minimum of 0.982 (for 4‐chlorodiphenyl ether) to a maximum of 0.999 (for 2 chlorophenol and 2 4 dichlorophenol). Minimum mean %recovery observed was 91.70 (for dimethyl phthalate) and a maximum %recovery was 111.38 (for pyrene), with a maximum %RSD of 14.28 in case of lindane. Studies indicate that the protocol is effective when analytes are strategically selected with their concentration, and ILIS were used for the quantification alongside in‐house verification of method parameters.

**TABLE 2:** Evaluation of method performance for quantitative analysis of multiclass analytes wastewater.

Sr. No.	Name of compound (compounds with *mark was quantified using IS)	Class of compound	RT	Ion ratio (± %RSD, *n* = 12)	*r* ^2^ (five point)	%Recovery at 1 ng/mL (TCDD at 0.05 ng/mL) (± %RSD *n* = 6)
					0.10–2.00 (TCDD @ 0.01–0.1) ng/mL	
1	Nitrobenzene*	VOCs	8.44	0.42 ± 6.73	0.992	96.28 ± 9.69
2	1 2 4 Trichlorobenzene*		8.96	0.59 ± 2.94	0.997	94.92 ± 7.83
3	Hexachlorobutadiene*		8.63	0.75 ± 1.72	0.995	97.30 ± 7.91
4	2 Chlorophenol*	Phenols	5.98	0.35 ± 1.41	0.999	100.38 ± 2.30
5	2 Nitrophenol*		8.87	0.64 ± 1.84	0.998	102.25 ± 3.05
6	Phenol*		5.94	0.19 ± 9.36	0.997	99.35 ± 7.70
7	2 4 Dichlorophenol*		8.94	0.53 ± 2.45	0.999	98.25 ± 4.23
8	4‐Chlorodiphenyl ether	Other chemicals	12.77	0.79 ± 3.94	0.982	96.07 ± 5.91
9	2 6 Dinitrotoluene		12.41	0.26 ± 8.95	0.994	96.93 ± 8.96
10	4‐Bromodiphenyl ether		13.48	0.87 ± 3.14	0.989	96.20 ± 8.04
11	Dimethyl Phthalate*	Phthalate	12.05	0.40 ± 3.80	0.995	91.70 ± 6.24
12	Fluorene*	PAHs	12.93	0.05 ± 7.58	0.997	109.35 ± 5.74
13	Naphthalene*		9.39	0.55 ± 4.04	0.988	92.47 ± 4.52
14	Acenaphthene*		12.24	0.19 ± 2.39	0.997	105.35 ± 2.77
15	Pyrene*		16.33	0.12 ± 9.40	0.983	111.38 ± 6.17
16	Hexachlorobenzene	OCPs and POPs	13.46	0.20 ± 6.07	0.992	91.80 ± 8.05
17	Heptachlor		10.11	0.01 ± 12.13	0.996	107.72 ± 2.94
18	Lindane		14.45	0.82 ± 2.27	0.996	94.13 ± 14.28
19	Chlordane		15.62	0.16 ± 3.54	0.986	108.48 ± 13.99
20	2 3 7 8 TCDD*		16.95	0.85 ± 4.86	0.989	105.34 ± 10.89

### 3.3 Method Greenness and Its Practicality

**FIGURE 4:**
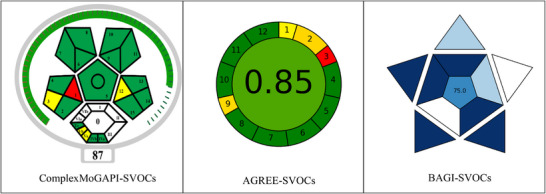
Evaluation of method sustainability and practicality by MoGAPI, AGREE, and BAGI.

The Green Analytical Procedure Index (GAPI) offers a visual assessment of the environmental impact and the overall sustainability of analytical methods. In 2021, ComplexGAPI enhanced this tool with the addition of hexagonal segment to assess activities beyond sample preparation and final analysis. This enhanced version of GAPI broadens sustainability evaluations but lacks a unified scoring system for individual methods. To address this issue, the next‐generation software ComplexMoGAPI was proposed to streamline the assessment process through a scoring system. GAPI's color code visually rates the environmental impact of analytical methods: green for low impact (high sustainability), orange for moderate, and red for high impact (low sustainability). This color‐coding system helps users quickly evaluate the environmental impact of analytical methods and choose procedures accordingly [36, 37, 38]. In this system, a score of 100 indicates that the method is fully green and excellent if it exceeds 75, acceptable if it is greater than 50, and not acceptable if it falls below 50. Parameters such as purification (VIa and VIb) and E‐factor are not applicable for the reported method and were excluded from the evaluations of ComplexMoGAPI, as in Figure 4 [26]. Combining pentagram and hexagram assessments, ComplexMoGAPI proved that the proposed method meets sustainability criteria, resulting in a total eco‐friendly score of 87 [https://mostwiedzy.pl/en/justyna‐plotka‐wasylka,647762‐1/complexgapi, https://bit.ly/ComplexMoGAPI].

The AGREE, an analytical greenness calculator uses a clock‐like graph displaying the overall score and color at the center. This graph shows 12 sections representing GAC principles. Each section is rated from zero to one, with one indicating adherence to green practices, and zero indicating non‐adherence, marked in red. The score in the middle of the pictogram shows eco‐friendliness, with values near 1 and dark green indicating a greener procedure [27, 39]. As illustrated in Figure 4, eight principles of the reported method comply with the greenness requirement. The third principle fails to meet the requirement due to offline sample preparation, and the first, second, and ninth principles partially meet it. However, the proposed method aligns with sustainability criteria and greenness score from AGREE, achieving an overall eco‐friendly score of 0.85 [https://mostwiedzy.pl/en/wojciech‐wojnowski,174235‐1/AGREE].

The BAGI complements established greenness evaluation metrics and gives a quick overview of strengths and weaknesses of methods regarding its practicality in general laboratory settings. The evaluation covers ten key attributes: the type of analysis, the number of analytes assessed per method, the simultaneous analysis of multiple analytes, the analytical technique used, the sample analysis rate per hour, the level of automation, the reagents and materials used, the instrument type, the sample preparation process, and the processing volume. These inputs are utilized to generate an asteroid‐shaped pictogram for the proposed method, with scores ranging from 25 to 100 [28, 40]. A high score of 75.0 signifies greater practicality of the reported method, as illustrated in Figure 4 (https://mostwiedzy.pl/en/justyna‐plotka‐wasylka,647762‐1/BAGI).

## 4 Conclusion

The automated DH‐RTC‐PAL‐SPME Arrow method combined with APGC‐MS/MS detection was found to be effective for analyzing semi volatile organic pollutants in wastewater. The integration of high‐throughput sample preparation and detection employing the advanced model of APGC‐MS/MS has demonstrated the presence of quasi‐molecular ions in the MS spectra of most analytes. This proves improved selectivity of the method for isomeric and close eluting pollutants, when compared to the GC‐MS with EI source of ionization. The analytes were separated by gas chromatography in 24 min, plus an extra 4 min for column conditioning. In contrast, the traditional method takes 50 min of GC run time for wastewater analysis [1]. Initial performance of the SPME Arrow was extensively evaluated (*n* = 35) by continuous injections on Days 1 and 2, with the satisfactory results for inter‐day and intra‐day precision. The method verification began by evaluating eight molecules from different classes of GC‐compatible pollutants, showing that the technique is effective for analyzing various types of components. Thereafter, the method was evaluated for analysis of wastewater for priority multiclass pollutants as listed in USEPA625.1/2026, with and without addition of the salt. Based on the responses and %RSDs, it can be recommended to proceed without the addition of salt and analyze all analytes in a single run. The method proves to be effective for monitoring various water types (from clean ASTM Type 1 water to complex wastewater) for semi volatile organic pollutants. Following internal verification, tests using specific analytes at lower concentrations and ILIS for quantification demonstrated that the protocol is suitable for quantitative analysis, provided that method parameters, analytes, IS, and concentrations are carefully optimized. The method has the potential to achieve LODs and LOQs in lower parts per trillion through extensive evaluation and optimization of SPME Arrow extraction parameters, including the choice of SPME Arrow (phase and coating thickness), analyte compatibility, sample conditions (volume, pH, matrix effects, ionic strength), penetration depths (vial and injector), extraction dynamics (agitation speed, stirring), time–temperature factors (incubation, extraction, desorption), and injection mode (split/split less). This automated method has achieved high scores in greenness evaluations (ComplexMoGAPI, AGREE) and have received an impressive BAGI score for their practicality and environmental sustainability. The proposed method meets the requirements of sustainable analytical chemistry discussed in many recently published research articles [41, 42, 43, 44, 45, 46, 47, 48]. The integration of high‐throughput sample preparation and detection has shown significant improvements in selectivity, precision and efficiency compared to traditional methods. The automated sample preparation method using DH‐RTC‐PAL‐SPME Arrow and APGC‐MS/MS detection has proven effective for analyzing multiclass pollutants in wastewater.

## Author Contributions

**Dnyaneshwar Shinde**: visualization, investigation, conceptualization, writing – original draft, validation, supervision, software, methodology, formal analysis, data curation. **Urvikkumar Dhagat**: validation, software, methodology, formal analysis, review and editing, data curation. **Vijayakumar Murugan**: validation, software, methodology, formal analysis, review and editing, data curation. **Parth Gupta**: methodology, formal analysis, review and editing, visualization. **Raghu Tadala**: methodology, formal analysis, review and editing, visualization. **Bhaskar Karubothula**: methodology, formal analysis, review and editing, visualization. **Chaitanya Devireddy**: software, review and editing. **Edward Stanislaus**: software, review and editing. **Samara Bin Salem**: visualization, supervision, resources. **Wael Elamin**: visualization, supervision, resources, project administration. **Grzegorz Brudecki**: writing – review and editing, visualization, validation, supervision, resources, project administration, methodology, formal analysis, data curation, conceptualization.

## Conflicts of Interest

The authors declare no conflicts of interest.

## Supporting information




**Supporting File 1**: jssc70328‐sup‐0001‐SuppMat.docx.

## Data Availability

Data will be made available on request.

## References

[1] USEPA , “Method 625.1: Base/Neutrals and Acids by GC/MS,” 2016, https://www.epa.gov/sites/default/files/2017‐08/documents/method_625‐1_2016.pdf.

[2] USEPA , “Method 3510C Separatory Funnel Liquid‐Liquid Extraction,” 1996, https://www.epa.gov/sites/default/files/2015‐12/documents/3510c.pdf.

[3] USEPA , “Method 8100 Polynuclear Aromatic Hydrocarbons,” 1986, https://www.epa.gov/sites/default/files/2015‐12/documents/8100.pdf.

[4] USEPA , “Method 8410 Gas Chromatography/Fourier Transform Infrared Spectrometry for Semi Volatile Organics: Capillary Column,” 2014, https://www.epa.gov/sites/default/files/2015‐12/documents/8410.pdf.

[5] USEPA , “Method 8270E Semi Volatile Organic Compounds by Gas Chromatography/Mass Spectrometry,” 2018, https://www.epa.gov/sites/default/files/2020‐10/documents/method_8270e_update_vi_06‐2018_0.pdf.

[6] USEPA , “Method 610: Polynuclear Aromatic Hydrocarbons, Appendix A To Part 136 Methods for Organic Chemical Analysis of Municipal and Industrial Wastewater,” 1984, https://www.epa.gov/sites/default/files/2015‐10/documents/method_610_1984.pdf.

[7] USEPA , “Method 8310 Polynuclear Aromatic Hydrocarbons,” 1986, https://www.epa.gov/sites/default/files/2015‐12/documents/8310.pdf.

[8] USEPA , “Method 525.3 Determination of Semi Volatile Organic Chemicals in Drinking Water by Solid Phase Extraction and Capillary Column and GC/MS,” 2012, https://cfpub.epa.gov/si/si_public_record_report.cfm?Lab=NERL&dirEntryId=241188.

[9] USEPA , “Method 1625C Semi Volatile Organic Compounds by Isotope Dilution GCMS,” 1989, https://www.epa.gov/sites/default/files/2015‐09/documents/method_1625c_1989.pdf.

[10] APHA , “Method 6410B, Liquid‐Liquid Extraction Gas Chromatographic/Mass Spectrometric Method,” 2000, 10.2105/SMWW.2882.124.

[11] International Standard , “ISO 7981‐2:2005(E) Water Quality—Determination of Polycyclic Aromatic Hydrocarbons (PAH). ISO.Org,” 2005, https://www.iso.org/standard/33884.html.

[12] International Standard , “ISO 17993 Water Quality—Determination of 15 Polycyclic Aromatic Hydrocarbons (PAH) in Water by HPLC With Fluorescence Detection After Liquid‐Liquid Extraction. ISO.Org,” 2002, https://www.iso.org/standard/31666.html.

[13] American Society for Testing and Materials , Standard Test Method for Quantification of Complex Polycyclic Aromatic Hydrocarbon Mixtures or Petroleum Oils in Water (American Society for Testing and Materials, 2024).

[14] USEPA , “Administration Finalizes Ban on Most Uses of Methylene Chloride, Protecting Workers and Communities From Fatal Exposure,” 2024, https://www.epa.gov/newsreleases/biden‐harris‐administration‐finalizes‐ban‐most‐uses‐methylene‐chloride‐protecting.

[15] D. Shinde , V. Murugan , U. Dhagat , et al., “Introduction of a Small Volume Ethyl Acetate Based Liquid‐Liquid Extraction Procedure for Analysis of Polycyclic Aromatic Hydrocarbons in Wastewater by Atmospheric Pressure Gas Chromatography‐Mass Spectrometry and Evaluation of Method Greenness,” Journal of Chromatography A 1740 (2025): 465563, https://pubmed.ncbi.nlm.nih.gov/39642662/. 39642662 10.1016/j.chroma.2024.465563

[16] J. Pawliszyn , Solid‐Phase Microextraction in Perspective (Elsevier, 2011), 1–12.

[17] J. S. Herrington , G. A. Gómez‐Ríos , C. Myers , G. Stidsen , and D. S. Bell , “Hunting Molecules in Complex Matrices With SPME Arrows: A Review,” Separations 7, no. 1 (2020): 12, 10.3390/separations7010012.

[18] L. Setkova , S. Risticevic , C. M. Linton , G. Ouyang , L. M. Bragg , and J. Pawliszyn , “Solid‐Phase Microextraction–Gas Chromatography–Time‐of‐Flight Mass Spectrometry Utilized for the Evaluation of the New‐Generation Super Elastic Fiber Assemblies,” Analytica Chimica Acta 581, no. 2 (2007): 221–231, 10.1016/j.aca.2006.08.022. 17386448

[19] M. Ziegler and H. G. Schmarr , “Comparison of Solid‐Phase Microextraction Using Classical Fibers Versus Mini‐Arrows Applying Multiple Headspace Extraction and Various Agitation Techniques,” Chromatographia 82, no. 2 (2018): 635–640, 10.1007/s10337-018-3659-1.

[20] J. Herrington , “SPME Arrow Blog 7 ChromaBLOGraphy: Restek | Chromatography Products and Solutions,” 2025, https://www.youtube.com/watch?v=shQU_JMetVY.

[21] J. Herrington , “SPME Arrow Blog 2 ChromaBLOGraphy: Restek's Chromatography Blog,” Accessed April 19, 2025, https://blog.restek.com/?p=37889.

[22] G. A. Gómez‐Ríos , N. Reyes‐Garcés , and J. Pawliszyn , “Evaluation of a Multi‐Fiber Exchange Solid‐Phase Microextraction System and Its Application to On‐Site Sampling,” Journal of Separation Science 38, no. 20 (2015): 3560–3567, 10.1002/jssc.201500158. 26311558

[23] S. S. Cai , J. A. Syage , K. A. Hanold , and M. P. Balogh , “Ultra Performance Liquid Chromatography−Atmospheric Pressure Photoionization‐Tandem Mass Spectrometry for High‐Sensitivity and High‐Throughput Analysis of U.S. Environmental Protection Agency 16 Priority Pollutants Polynuclear Aromatic Hydrocarbons,” Analytical Chemistry 81, no. 6 (2009): 2123–2128. 19227980 10.1021/ac802275e

[24] T. Portolés , S. J. M. Johannes , and F. Hernández , “Advantages of Atmospheric Pressure Chemical Ionization in Gas Chromatography Tandem Mass Spectrometry: Pyrethroid Insecticides as a Case Study,” Analytical Chemistry 84, no. 22 (2012): 9802–9810, 10.1021/ac301699c. 23006011

[25] L. Cherta , T. Portolés , J. Beltran , E. Pitarch , J. G. J. Mol , and F. Hernández , “Application of Gas Chromatography–(Triple Quadrupole) Mass Spectrometry With Atmospheric Pressure Chemical Ionization for the Determination of Multiclass Pesticides in Fruits and Vegetables,” Journal of Chromatography A 1314 (2013): 224–240, 10.1016/j.chroma.2013.09.029. 24070626

[26] F. R. Mansour , K. M. Omer , and J. Płotka‐Wasylka , “A Total Scoring System and Software for Complex Modified GAPI (ComplexMoGAPI) Application in the Assessment of Method Greenness,” Green Analytical Chemistry 10 (2024): 100126.

[27] F. Pena‐Pereira , W. Wojnowski , and M. Tobiszewski , “AGREE—Analytical GREEnness Metric Approach and Software,” Analytical Chemistry 92, no. 14 (2020): 10076–10082, 10.1021/acs.analchem.0c01887. 32538619 PMC7588019

[28] N. Manousi , W. Wojnowski , J. Płotka‐Wasylka , and V. Samanidou , “Blue Applicability Grade Index (BAGI) and Software: A New Tool for the Evaluation of Method Practicality,” Green Chemistry 25, no. 19 (2023): 7598–7604, 10.1039/D3GC02347H.

[29] N. Aldegunde‐Louzao , M. Lolo‐Aira , and C. Herrero‐Latorre , “Fast Phthalate Detection in Textile Samples: A LC‐MS/MS Screening Method Using Precursor Ion Scans,” chemistry proceedings (2024): 1–1, 10.3390/ecsoc-28-20150.

[30] S. Löbbecke , A. Pape , L. Montero , F. Uteschil , J. F. Ayala‐Cabrera , and O. J. Schmitz , “Improving the Reliability of Phthalate Esters Analysis in Water Samples by Gas Chromatography‐Tube Plasma Ionization‐High‐Resolution Mass Spectrometry (GC‐TPI‐HRMS),” Talanta 285 (2024): 127388–127388, 10.1016/j.talanta.2024.127388. 39700716

[31] X. Wang , X. Sun , X. Wang , et al., “Determination of 15 Phthalic Acid Esters Based on GC–MS/MS Coupled With Modified QuEChERS in Edible Oils,” Food Chemistry: X 16 (2022): 100520, 10.1016/j.fochx.2022.100520. 36519086 PMC9743266

[32] SANTE 11312/2021 v2 , “Analytical Quality Control and Method Validation Procedures for Pesticide Residues Analysis in Food and Feed, EURL for Residues of Pesticides,” 2024, http://food.ec.europa.eu/system/files/2023‐11/pesticides_mrl_guidelines_wrkdoc_2021‐11312.pdf.

[33] European Pharmacopeia , “Chromatography—Notice of Adoption of Harmonized Standard,” Accessed April 24, 2025, https://www.usp.org/harmonization‐standards/pdg/excipients/chromatography.

[34] L. C. Martins , M. S. M. S. F. Acevedo , M. R. Gama , and F. R. P. Rocha , “Salt‐Assisted Liquid‐Liquid Extraction and On‐Column Concentration for Chromatographic Determination of Phenolic Compounds in Beer,” Advances in Sample Preparation 9 (2024): 100107, 10.1016/j.sampre.2024.100107.

[35] T. Gezahegn , B. Tegegne , F. Zewge , and B. S. Chandravanshi , “Salting‐Out Assisted Liquid–Liquid Extraction for the Determination of Ciprofloxacin Residues in Water Samples by High Performance Liquid Chromatography–Diode Array Detector,” BMC Chemistry 13, no. 1 (2019): 28, 10.1186/s13065-019-0543-5. 31384776 PMC6661818

[36] N. M. Abdulhussein , N. M. Muslim , M. A. Hussien , E. A. Azooz , and E. A. J. Al‐Mulla , “Green Preconcentration Procedures for the Determination of Aluminium in Bottled Beverages Prior to Electrothermal Atomic Absorption Spectroscopy: A Comparative Study With Environmental Assessment Tools,” Journal of the Iranian Chemical Society 21, no. 5 (2024): 1203–1212, 10.1007/s13738-024-02979-y.

[37] N. M. Muslim , B. K. Hussain , A. N. Mahmood , and A. E. Adnan , “Determination of Selenium in Black Tea Leaves Using the Air‐Assisted Cloud Point Extraction Method: Evaluation of the Method's Environmental Performance,” Analytical and Bioanalytical Chemistry Research 11, no. 1 (2024): 11–22.

[38] F. A. Semysim , B. K. Hussain , M. A. Hussien , E. A. Azooz , and D. Snigur , “Assessing the Greenness and Environmental Friendliness of Analytical Methods: Modern Approaches and Recent Computational Programs,” Critical Reviews in Analytical Chemistry 55 (2024): 670–683, 10.1080/10408347.2024.2304552. 38241068

[39] D. Moema , T. A. Makwakwa , B. E. Gebreyohannes , S. Dube , and M. M. Nindi , “Hollow Fiber Liquid Phase Microextraction of Fluoroquinolones in Chicken Livers Followed by High Pressure Liquid Chromatography: Greenness Assessment Using National Environmental Methods Index Label (NEMI), Green Analytical Procedure Index (GAPI), Analytical GREEnness Metric (AGREE), and Eco Scale,” Journal of Food Composition and Analysis 117 (2023): 105131.

[40] R. Mahdavi , Z. Talebpour , and M. Noori , “Application of New Green Evaluation Tools to Assess the Environmental Impact of Analytical Procedures Based on Solid Phase Microextraction Techniques,” Green Analytical Chemistry 10 (2024): 100144.

[41] S. S. Nasrollahi and Y. Yamini , “Green Dispersive Solid‐Phase Microextraction of Melamine Using Crosslinked Beta‐Cyclodextrin With Citric Acid Followed by High‐Performance Liquid Chromatography,” Journal of Separation Science 46, no. 14 (2023): e2300132, 10.1002/jssc.202300132. 37232223

[42] A. Doumtsi , N. Manousi , C. Karavasili , D. G. Fatouros , P. D. Tzanavaras , and C. K. Zacharis , “A Simple and Green Liquid Chromatography Method for the Determination of Ibuprofen in Milk‐Containing Simulated Gastrointestinal Media for Monitoring the Dissolution Studies of Three‐Dimensional‐Printed Formulations,” Journal of Separation Science 45, no. 21 (2022): 3955–3965, 10.1002/jssc.202200444. 36054076

[43] M. Pasham , N. V. V. D. P. Boppy , N. Vadagam , S. B. Haridasyam , and N. S. Lakka , “Development and Validation of an Eco‐Friendly HPLC Method for the Quantification of Organic Impurities in Flecainide Acetate Bulk and Tablets: A Green Chemistry Assessment,” Separation Science Plus 8, no. 9 (2025), 10.1002/sscp.70127.

[44] V. D. Torgal , V. Mastiholimath , and R. Koli , “Development of a Stability‐Indicating RP‐HPLC Method for Pioglitazone in Cubosomal and Biological Matrices: A Quality by Design‐Driven, Lean Six Sigma, and Green Chemistry Approach,” Separation Science Plus 8, no. 5 (2025), 10.1002/sscp.70055.

[45] B. Karubothula , V. Cheerala , D. Shinde , et al., “Automation in wastewater‐based epidemiology for efficient analysis of 123 multi‐class illicit drugs: evaluating method eco‐friendliness and practicality,” Microchemical Journal 218, (2025) 115270, 10.1016/j.microc.2025.115270.

[46] B. Karubothula , V. V. Kota , D. Shinde , et al., “Next‐Generation Wastewater‐Based Epidemiology: Green Automation for Detecting 69 Multiclass Pharmaceutical and Personal Care Products in Wastewater Using 96‐Well Plate Solid‐Phase Extraction by LC‐MS/MS,” Molecules 30, no. 18 (2025) 3694, 10.3390/molecules30183694. 41011589 PMC12472227

[47] B. Karubothula , C. Devireddy , D. Shinde , et al., “Advancing Wastewater Surveillance: Development of High‐Throughput Green Robotic SPE‐UPLC‐MS/MS Workflow for Monitoring of 27 Steroids and Hormones,” Applied Sciences 15, no. 18 (2025) 10012, 10.3390/app151810012.

[48] D. Shinde , B. Karubothula , P. Gupta , et al., Advances in the Analysis of Contaminants, Illicit Drugs, Pharmaceuticals, and Hormones for Wastewater‐Based Epidemiology (WBE) Using Automated Extraction and Detection by LC and GC‐MS/MS: Evaluation of Method Eco‐Friendliness and Practicality. Mass Spectrometry ‐ Applications and Recent Advances [Working Title], (2025), 10.5772/intechopen.1011550.

