# Supplementary material for: Automated Analysis of Pollutants in Wastewater Using Atmospheric Pressure Gas Chromatography‐Tandem Quadrupole Mass Spectrometer With Robotic Autosampler and Solid Phase Micro Extraction
Source: J Sep Sci. 2025 Nov 25;48(11):e70328. doi: 10.1002/jssc.70328 (PMC12645424; doi:10.1002/jssc.70328)
Supplement: Supplementary file 1 — Supporting File 1: jssc70328‐sup‐0001‐SuppMat.docx. [file JSSC-48-e70328-s001.docx]

**Supplementary materials**

**Automated Analysis of Pollutants in Wastewater Using Atmospheric Pressure Gas Chromatography-Tandem Quadrupole Mass Spectrometer with Robotic Autosampler and Solid Phase Micro Extraction**

Dnyaneshwar Shinde^*1^, Urvikkumar Dhagat^1^, Vijayakumar Murugan^1^, Parth Gupta^1^, Raghu Tadala^1^, Bhaskar Karubothula^1^, Chaitanya Devireddy^1^, Edward Stanislaus^1^, Samara Bin Salem^2^, Wael Elamin^1^ and Grzegorz Brudecki^1^

^1^RASID Laboratory of Abu Dhabi Quality & Conformity Council (ADQCC) & M42 Environmental Sciences, Abu Dhabi P.O. Box 853, United Arab Emirates

^2^Abu Dhabi Quality and Conformity Council UAE

*Correspondence: Dnyaneshwar Shinde ([dshinde@m42.ae](mailto:dshinde@m42.ae))

(a)

(b)

Table 2: MRM method for targeted Pollutants and Internal Standards (IS)

| **Sr. No.** | **Compound Name**  **(Molecular Weight, in Da)** | **Details of APGC-MS/MS Method** | | | | | | | | |
| --- | --- | --- | --- | --- | --- | --- | --- | --- | --- | --- |
|  |  | **RT (in min.)** | **MRM Transitions** | | **CV (V)** | **CE  (Q1, Q2) (eV)** | **Ion Ratio ±**  **% RSD** | **Details of the IS used for Quantification** | | |
|  |  |  | **Parent Ion** | **Quantifier, Qualifier (Q1, Q2)** |  |  |  | **Name of IS** | **MRM Transition (Q1)** | **CV, CE  (V, eV)** |
| **Poly-Aromatic Hydrocarbons (PAHs)** | | | | | | | | | | |
| 1 | Naphthalene (128) | 8.57 | 128.0 | 102.0, 78.0 | 20 | 20, 10 | 0.660 ± 3.63 | Naphthalene d8 | 136.00 > 108.00 | 55, 20 |
| 2 | 2-Chloronaphthalene (162) | 11.05 | 161.9 | 127.0, 77.0 | 30 | 25, 35 | 0.172 ± 6.72 | 2-Chloronaphthalene d7 | 169.00 > 134.00 | 20, 30 |
| 3 | Acenaphthylene (152) | 11.38 | 152.0 | 152.0, 151.0 | 65 | 10, 28 | 0.413 ± 15.96 | Acenaphthylene d8 | 160.00 > 158.00 | 65, 28 |
| 4 | Acenaphthene (154) | 11.56 | 154.0 | 153.0, 152.0 | 40 | 20, 30 | 0.090 ± 17.94 | Acenaphthene d10 | 164.00 > 162.00 | 40, 20 |
| 5 | Fluorene (166) | 12.26 | 166.0 | 165.0, 164.0 | 35 | 20, 35 | 0.053 ± 12.25 | Fluorene d10 | 176.00 > 174.00 | 35, 20 |
| 6 | Phenanthrene (178) | 13.67 | 178.0 | 177.0. 152.0 | 65 | 25, 30 | 0.778 ± 16.68 | Phenanthrene d10 | 188.00 >186.00 | 65, 25 |
| 7 | Anthracene (178) | 13.72 | 178.0 | 177.0. 152.0 | 65 | 25, 30 | 0.628 ± 25.43 | Anthracene d10 | 188.00 > 186.00 | 65, 25 |
| 8 | Fluoranthene (202) | 15.27 | 202.0 | 201.0, 200.0 | 70 | 35, 35 | 0.843 ± 13.25 | Fluoranthene d10 | 212.00 > 210.00 | 70, 35 |
| 9 | Pyrene (202) | 15.63 | 202.0 | 201.0, 200.0 | 70 | 35, 35 | 0.496 ± 16.78 | Pyrene d10 | 212.00 > 210.00 | 70, 35 |
| 10 | Benzo(a)anthracene (228) | 17.17 | 228.0 | 228.0, 226.0 | 30 | 15, 30 | 0.181 ± 29.97 | Benz a anthracene d12 | 240.00 > 240.00 | 30, 15 |
| 11 | Chrysene (228) | 17.27 | 228.0 | 228.0, 226.0 | 30 | 15, 30 | 0.218 ± 21.69 | Chrysene d12 | 240.00 > 240.00 | 30, 15 |
| 12 | Benzo(b)fluoranthene (252) | 18.58 | 253.1 | 252.1, 250.1 | 40 | 35, 52 | 0.269 ± 9.44 | Benzo(b)fluoranthene d12 | 264.00 > 236.00 | 30, 40 |
| 13 | Benzo(k)fluoranthene (252) | 18.62 | 253.1 | 252.1, 250.1 | 40 | 35, 52 | 0.250 ± 8.85 | Benzo(k)fluoranthene d12 | 264.00 > 236.00 | 30, 40 |
| 14 | Benzo(a)pyrene (252) | 19.22 | 253.1 | 252.1, 250.1 | 40 | 35, 52 | 0.218 ± 6.58 | Benzo a pyrene d12 | 264.00 > 264.00 | 30, 15 |
| 15 | Dibenz(a,h)anthracene (278) | 22.41 | 278.0 | 278.0, 276.0 | 40 | 15, 30 | 19.033 ± 7.18 | Dibenz(a,h)anthracene d14 | 293.00 > 291.50 | 10, 40 |
| 16 | Indeno (1,2,3-cd) pyrene (276) | 22.48 | 276.0 | 276.0, 274.0 | 40 | 15, 40 | 0.202 ± 4.40 | Indeno (1,2,3-cd) pyrene d12 | 288.00 > 284.19 | 40,50 |
| 17 | Benzo(ghi)perylene (276) | 23.58 | 276.0 | 276.0, 274.0 | 40 | 15, 40 | 0.139 ± 4.67 | Benzo(ghi)perylene d12 | 288.00 > 284.26 | 40, 40 |
| **Organo-Chlorine Pesticides (OCPs)** | | | | | | | | | | |
| 18 | alpha-BHC (288) | 12.95 | 219.01 | 182.74, 144.82 | 20 | 10, 20 | 0.703 ± 14.98 | NA | NA | NA |
| 19 | gamma-BHC (288) | 13.43 | 219.01 | 182.74, 144.82 | 20 | 10, 20 | 0.440 ± 25.84 | NA | NA | NA |
| 20 | Heptachlor (370) | 13.59 | 271.83 | 236.91, 142.65 | 20 | 20, 40 | 0.138 ± 5.04 | NA | NA | NA |
| 21 | beta-BHC (288) | 13.80 | 219.01 | 182.74, 144.82 | 20 | 10, 20 | 0.640 ± 27.88 | NA | NA | NA |
| 22 | delta-BHC (288) | 14.08 | 219.01 | 182.74, 144.82 | 20 | 10, 20 | 0.594 ± 22.31 | NA | NA | NA |
| 23 | Aldrin (362) | 14.49 | 292.98 | 185.94, 257.98 | 20 | 20, 10 | 0.643 ± 10.46 | NA | NA | NA |
| 24 | Heptachlor epoxide (386) | 14.71 | 352.87 | 262.94, 316.84 | 20 | 20, 10 | 0.785 ± 11.12 | NA | NA | NA |
| 25 | Chlordane cis (406) | 14.95 | 372.93 | 265.83, 232.02 | 20 | 20, 40 | 0.424 ± 19.42 | NA | NA | NA |
| 26 | Chlordane trans (406) | 15.04 | 372.93 | 265.83, 232.02 | 20 | 20, 40 | 0.462 ± 24.68 | NA | NA | NA |
| 27 | alpha-endosulfan (404) | 15.12 | 338.94 | 266.95, 159.73 | 20 | 20, 20 | 0.708 ± 13.34 | NA | NA | NA |
| 28 | 4,4'-DDE (316) | 15.28 | 316.00 | 245.96, 247.96 | 20 | 30, 30 | 0.748 ± 16.81 | NA | NA | NA |
| 29 | Dieldrin (378) | 15.43 | 345.00 | 262.62, 232.02 | 20 | 20, 30 | 0.621 ± 14.74 | NA | NA | NA |
| 30 | Endrin (378) | 15.78 | 380.00 | 344.86, 281.04 | 20 | 10, 10 | 0.309 ± 19.32 | NA | NA | NA |
| 31 | 4,4'-DDD (318) | 15.91 | 234.86 | 164.86, 199.00 | 20 | 20, 30 | 0.035 ± 26.84 | NA | NA | NA |
| 32 | beta-endosulfan (404) | 16.09 | 338.94 | 266.71, 158.77 | 20 | 10, 20 | 1.430 ± 17.69 | NA | NA | NA |
| 33 | 4,4'-DDT (352) | 16.16 | 235.14 | 164.90, 198.93 | 20 | 20, 20 | 0.260 ± 11.63 | NA | NA | NA |
| 34 | Endrin aldehyde (378) | 16.33 | 345.02 | 245.08, 278.8 | 20 | 20, 10 | 1.023 ± 18.90 | NA | NA | NA |
| 35 | Endosulfan sulfate (420) | 16.52 | 421.86 | 386.89, 226.97 | 20 | 10, 20 | 0.556 ± 30.29 | NA | NA | NA |
| **Phthalates Esters (PAEs)** | | | | | | | | | | |
| 36 | Dimethyl phthalate (194) | 11.42 | 163.05 | 77.05, 135.05 | 30 | 20, 13 | 0.350 ± 11.86 | Dimethyl phthalate d3 | 166.44 > 77.92 | 20, 20 |
| 37 | Diethyl phthalate (222) | 12.54 | 223.1 | 149.05, 177.05 | 20 | 15, 5 | 1.182 ± 4.03 | Diethyl phthalate d14 | 137.10 > 186.00 | 5,10 |
| 38 | Di-n-butyl phthalate (278) | 14.34 | 279.15 | 205.10, 149.05 | 30 | 5, 5 | 1.478 ± 2.50 | Di-n-butyl phthalate d22 | 301.00 > 218.00 | 30, 10 |
| 39 | Benzyl butyl phthalate (312) | 16.15 | 313.2 | 91.05, 149.05 | 30 | 12, 12 | 0.650 ± 14.16 | Benzyl butyl phthalate d4 | 317.00 > 91.00 | 30, 12 |
| 40 | Bis 2 ethylhexyl phthalate (390) | 16.53 | 391.2 | 149.05, 167.10 | 30 | 17, 11 | 0.146 ± 6.10 | Bis 2 ethylhexyl phthalate d4 | 395.00 > 171.00 | 30, 10 |
| 41 | Di-n-octyl phthalate (390) | 17.36 | 391.2 | 149.05, 261.10 | 30 | 17, 6 | 0.139 ± 2.98 | Di-n-octyl phthalate d4 | 395.00 > 153.00 | 50, 10 |
| **Phenols** | | | | | | | | | | |
| 42 | Phenol (94) | 5.57 | 94.05 | 66.05, 65.05 | 20 | 12, 15 | 0.195 ± 9.17 | Phenol d5 | 99.30 > 71.03 | 20, 10 |
| 43 | 2-Chlorophenol (128) | 5.61 | 127.9 | 63.90, 62.90 | 20 | 20, 15 | 0.346 ± 1.11 | 2-Chlorophenol d4 | 132.20 > 132.20 | 30, 5 |
| 44 | 2,4-Dimethylphenol (122) | 8.29 | 122.05 | 107.05, 77.05 | 30 | 12, 22 | 0.152 ± 2.24 | 2,4-Dimethylphenol d3 | 125.00 > 110.00 | 30, 20 |
| 45 | 2-Nitrophenol (139) | 8.55 | 139.5 | 109.05, 81.05 | 20 | 10, 12 | 0.841 ± 1.21 | 2 Nitrophenol d4 | 143.01 > 113.18 | 30, 10 |
| 46 | 2,4-Dichlorophenol (162) | 8.61 | 162.0 | 98.00, 126.00 | 30 | 15, 15 | 0.666 ± 2.41 | 2 4 Dichlorophenol d3 | 165.08 > 165.08 | 30, 5 |
| 47 | 4-Chloro-3-methylphenol (142) | 10.20 | 143.0 | 143.00, 142.00 | 40 | 3, 5 | 0.051 ± 4.25 | 4-Chloro-3-methylphenol d2 | 144.00 > 109.00 | 30, 20 |
| 48 | 2,4,6-Trichlorophenol (196) | 10.68 | 195.9 | 97.00, 132.00 | 30 | 30, 20 | 0.277 ± 4.85 | 2,4,6-Trichlorophenol-^13^C_6_ | 201.94 > 101.93 | 20, 30 |
| 49 | 4-Nitrophenol (139) | 12.49 | 139.05 | 109.05, 81.05 | 20 | 10, 12 | 0.373 ± 8.32 | 4 Nitrophenol d4 | 113.20 > 113.20 | 40, 5 |
| 50 | 2-Methyl-4,6-dinitrophenol (198) | 12.79 | 198.0 | 198.00, 167.00 | 10 | 5, 10 | NA | 2-Methyl-4,6-dinitrophenol d2 | 200.00 > 200.00 | 20, 2 |
| 51 | 2,4-Dinitrophenol (184) | 12.79 | 184.1 | 154.05, 107.05 | 30 | 8, 10 | 0.047 ± 35.68 | 2,4-Dinitrophenol d3 | 187.00 > 110.00 | 10, 10 |
| 52 | Pentachlorophenol (264) | 12.80 | 263.8 | 164.80, 166.70 | 30 | 32, 25 | 0.489 ± 10.30 | Pentachlorophenol-^13^C_6_ | 271.87 > 172.04 | 30, 30 |
| **Other Semi-Volatile Organic Compounds (Other SVOCs)** | | | | | | | | | | |
| 53 | Hexachloroethane (234) | 5.86 | 200.8 | 165.80, 163.80 | 30 | 15, 10 | 4.576 ± 14.46 | Hexachloroethane^13^C_1_ | 202.00 > 167.00 | 20, 20 |
| 54 | Bis 2 chloroisopropyl ether (170) | 5.92 | 93.02 | 93.02, 77.08 | 10 | 2, 2 | 0.943 ± 18.70 | NA | NA | NA |
| 55 | 1, 2 Dichlorobenzene (146) | 6.62 | 146.0 | 111.0, 113.0 | 35 | 30,30 | 0.099 ± 11.84 | 1, 4 Dichlorobenzene d4 | 149.95 > 115.01 | 30, 20 |
| 56 | N-nitrosodi-n-propylamine (130) | 7.27 | 131.05 | 43.05, 89.05 | 20 | 15, 5 | 1.546 ± 7.22 | N-nitrosodi-n-propylamine d14 | 126.00 > 78.00 | 20, 10 |
| 57 | Nitrobenzene (123) | 7.52 | 124.05 | 77.05, 94.05 | 20 | 12, 10 | 0.281 ± 2.71 | Nitrobenzene d5 | 129.19> 81.99 | 30, 20 |
| 58 | Hexachlorobutadiene (258) | 7.74 | 259.8 | 224.80, 222.80 | 30 | 15, 15 | 1.629 ± 6.70 | Hexachlorobutadiene ^13^C_4_ | 263.7 > 194.2 | 20, 30 |
| 59 | 1,2,4-Trichlorobenzene (180) | 8.09 | 179.9 | 144.90, 108.90 | 30 | 20, 30 | 0.657 ± 8.08 | 1,2,4-Trichlorobenzene d3 | 183.0 > 111.0 | 30, 30 |
| 60 | Octafluoronaphthalene (272) | 8.09 | 272.0 | 222.0 | 35 | 30 | NA | NA | NA | NA |
| 61 | Isophorone (138) | 8.15 | 139.05 | 69.05, 82.00 | 20 | 15, 3 | 0.094 ± 1.66 | Isophorone d8 | 147.0 > 47.0 | 10, 20 |
| 62 | Bis 2-chloroethoxy methane (172) | 8.21 | 64.91 | 64.91, 62.90 | 20 | 5, 5 | 13.679 ± 7.20 | Bis 2-chloroethoxy methane d8 | 181.0 > 67.0 | 20, 5 |
| 63 | 2,6-Dinitrotoluene (182) | 11.76 | 183.05 | 108.05, 91.05 | 30 | 10, 20 | 0.688 ± 4.78 | 2,6-Dinitrotoluene d3 | 186.00 > 186.00 | 20, 5 |
| 64 | 4-Chloro-di-phenyl ether (204) | 12.11 | 203.9 | 77.00, 141.00 | 30 | 30, 25 | 0.887 ± 4.21 | 4-Chloro-di-phenyl ether d5 | 210.00 > 210.00 | 30, 5 |
| 65 | 2,4-Dinitrotoluene (182) | 12.28 | 183.05 | 108.05, 91.05 | 30 | 10, 20 | 0.795 ± 9.03 | 2,4-Dinitrotoluene ^13^C_6_ | 188.07 > 125.00 | 20, 20 |
| 66 | Hexachlorobenzene (282) | 12.79 | 282.0 | 212.00, 213.70 | 40 | 30, 35 | 1.981 ± 8.28 | Hexachlorobenzene ^13^C_6_ | 289.87 > 255.08 | 20, 30 |
| 67 | 4-Bromo-di-phenyl ether (248) | 12.83 | 247.9 | 141.00, 77.00 | 30 | 25, 30 | 0.750 ± 10.83 | 4-Bromo-di-phenyl ether d5 | 254.00 > 175.00 | 30, 10 |
| 68 | Benzidine (184) | 15.12 | 184.0 | 184.00, 183.00 | 20 | 5, 5 | 0.192 ± 22.36 | Benzidine d8 | 193.00 > 174.00 | 10, 20 |
| 69 | 2,3,7,8 Tetrachlorodibenzodioxin (320) | 16.87 | 321.9 | 258.9, 256.9 | 35 | 30,30 | 0.100 ± 6.50 | NA | NA | NA |
| 70 | 3,3-Dichlorobenzidine (252) | 17.34 | 254.2 | 218.20, 154.10 | 30 | 28. 45 | 0.409 ± 39.83 | 3,3-Dichlorobenzidine d6 | 258.00 > 132.00 | 30, 50 |
| NA-Not applicable, Q1-Quantifier ion, Q2-Qualifier ion, CV-Cone Voltage, CE-Collision Energy, MRM-Multiple Residue Monitoring, RT-Retention Time, RSD- Relative Standard Deviation, IS- Internal Standard | | | | | | | | | | |

Table 3: Evaluation of SPME arrow extraction with and without salt addition

| **Sr. No.** | **Name of Compound** | **Results with Salt addition (n=6)** | | **Results without Salt addition (n=6)** | | **Increase in response on salt addition (folds)** | **QC Acceptance Criteria “s” (Standard deviation of DOC from EPA625.1/2016)** |
| --- | --- | --- | --- | --- | --- | --- | --- |
|  |  | **Mean Area** | **% RSD** | **Mean Area** | **% RSD** |  |  |
| **Poly-Aromatic Hydrocarbons (PAHs)** | | | | | | |  |
| 1 | Naphthalene | 25,545^a^ | 41.03 | 29,045^a^ | 32.62 | 0.88 | 39 |
| 2 | 2-Chloronaphthalene | 17,721,637^a^ | 32.06 | 23,271,039^a^ | 10.97 | 0.76 | 15 |
| 3 | Acenaphthylene | 42,116,035^a^ | 9.73 | 43,762,459^a^ | 2.61 | 0.96 | 45 |
| 4 | Acenaphthene | 38,738,305^a^ | 14.01 | 42,250,150^a^ | 2.60 | 0.92 | 29 |
| 5 | Fluorene | 41,801,688^a^ | 14.50 | 47,082,919^a^ | 3.17 | 0.89 | 23 |
| 6 | Phenanthrene | 15,064,723^a^ | 45.06 | 15,897,168^a^ | 11.13 | 0.95 | 24 |
| 7 | Anthracene | 8,828,236^a^ | 55.42 | 10,433,161^a^ | 9.89 | 0.85 | 40 |
| 8 | Fluoranthene | 5,971,086^a^ | 56.97 | 8,668,322^a^ | 21.78 | 0.69 | 40 |
| 9 | Pyrene | 5,466,744^a^ | 50.67 | 7,532,188^a^ | 19.64 | 0.73 | 30 |
| 10 | Benzo(a)anthracene | 6,064,672^a^ | 49.01 | 12326,834^b^ | 10.66 | 0.49 | 32 |
| 11 | Chrysene | 6,940,612^a^ | 53.72 | 12,188,265^b^ | 17.82 | 0.57 | 53 |
| 12 | Benzo(b)fluoranthene (sum) | 261,381^a^ | 50.66 | 551,644^b^ | 11.78 | 0.47 | 43 |
| 13 | Benzo(k)fluoranthene (sum) |  |  |  |  |  | 38 |
| 14 | Benzo(a)pyrene | 92,462^a^ | 49.30 | 174,141^b^ | 19.89 | 0.53 | 43 |
| 15 | Dibenz(a,h)anthracene | 301,428^a^ | 52.07 | 426,646^a^ | 52.76 | 0.71 | 75 |
| 16 | Indeno (1,2,3-cd) pyrene | 303,624^a^ | 51.63 | 464,930^a^ | 43.41 | 0.65 | 60 |
| 17 | Benzo(ghi)perylene | 291,242^a^ | 54.91 | 460,458^a^ | 48.54 | 0.63 | 61 |
| **Organo-Chlorine Pesticides (OCPs)** | | | | | | |  |
| 18 | alpha-BHC | 10,391,334^a^ | 52.08 | 4,588,114^b^ | 9.57 | 2.26 | NR |
| 19 | gamma-BHC | 7,607,157^a^ | 38.05 | 906,914^b^ | 10.09 | 8.39 | NR |
| 20 | Heptachlor | 16,705,445^a^ | 42.76 | 29,648,967^b^ | 18.43 | 0.56 | 44 |
| 21 | beta-BHC | 7,927,052^a^ | 49.61 | 2,462,893^b^ | 10.97 | 3.22 | 37 |
| 22 | delta-BHC | 3,083,445^a^ | 38.75 | 501,993^b^ | 11.53 | 6.14 | 77 |
| 23 | Aldrin | 769,838^a^ | 84.51 | 2,214,079^b^ | 37.48 | 0.35 | 39 |
| 24 | Heptachlor epoxide | 1,199,968^a^ | 90.63 | 1,985,535^a^ | 18.64 | 0.60 | 61 |
| 25 | Chlordane | 6,589^a^ | 65.27 | 19,957^b^ | 20.78 | 0.33 | NR |
| 26 | alpha-endosulfan | 563,906^a^ | 85.55 | 837,368^a^ | 11.38 | 0.67 | NR |
| 27 | 4,4'-DDE | 3,181,197^a^ | 78.00 | 12,521,608^b^ | 43.78 | 0.25 | 46 |
| 28 | Dieldrin | 206,555^a^ | 62.88 | 483,852^b^ | 20.21 | 0.43 | 38 |
| 29 | Endrin | 148,356^a^ | 75.98 | 291,283^b^ | 34.76 | 0.51 | NR |
| 30 | 4,4'-DDD | 3,266,113^a^ | 67.35 | 8,521,189^b^ | 34.96 | 0.38 | 56 |
| 31 | beta-endosulfan | 173,262^a^ | 43.66 | 151,957^a^ | 34.00 | 1.14 | NR |
| 32 | 4,4'-DDT | 1,201,493^a^ | 77.81 | 4,607,610^b^ | 46.39 | 0.26 | 81 |
| 33 | Endrin aldehyde | 112,114^a^ | 60.91 | 157,335^a^ | 37.62 | 0.71 | 45 |
| 34 | Endosulfan sulfate | 66,929^a^ | 47.84 | 30,227^b^ | 33.64 | 2.21 | 42 |
| **Phthalates Esters (PAEs)** | | | | | | |  |
| 35 | Dimethyl phthalate | 1,313,275^a^ | 31.80 | 50,610^b^ | 16.06 | 25.95 | 110 |
| 36 | Diethyl phthalate | 23,568,216^a^ | 35.43 | 1,742,096^b^ | 28.30 | 13.53 | 60 |
| 37 | Di-n-butyl phthalate | 30,833,800^a^ | 8.49 | 4,214,052^b^ | 22.19 | 7.32 | 28 |
| 38 | Benzyl butyl phthalate | 65,102^a^ | 53.13 | 38,711^a^ | 17.71 | 1.68 | 36 |
| 39 | Bis 2 ethylhexyl phthalate | 58,315^a^ | 67.99 | 93,493^a^ | 26.94 | 0.62 | 50 |
| 40 | Di-n-octyl phthalate | 7,730^a^ | 71.13 | 4,575^a^ | 15.82 | 1.69 | 42 |
| **Phenols** | | | | | | |  |
| 41 | Phenol | 15,103,907^a^ | 9.45 | 1,358,116^b^ | 17.78 | 11.12 | 39 |
| 42 | 2-Chlorophenol | 28,979,994^a^ | 13.18 | 3,417,845^b^ | 27.16 | 8.48 | 37 |
| 43 | 2,4-Dimethylphenol | 6,112,102^a^ | 19.93 | 443,218^b^ | 14.21 | 13.79 | 35 |
| 44 | 2-Nitrophenol | 20,042,645^a^ | 19.71 | 1,132,705^b^ | 32.55 | 17.69 | 33 |
| 45 | 2,4-Dichlorophenol | 47,521,250^a^ | 15.04 | 6,388,650^b^ | 15.99 | 7.44 | 30 |
| 46 | 4-Chloro-3-methylphenol | 8,760,402^a^ | 21.31 | 624,322^b^ | 5.11 | 14.03 | 44 |
| 47 | 2,4,6-Trichlorophenol | 33,031,378^a^ | 23.30 | 1,011,573^b^ | 16.63 | 32.65 | 35 |
| 48 | 4-Nitrophenol | 521,327^a^ | 24.67 | 27,301^b^ | 25.09 | 19.10 | 79 |
| 49 | 2-Methyl-4,6-dinitrophenol* | NA | NA | 1,295,636 | 38.49 | NA | 122 |
| 50 | 2,4-Dinitrophenol | 221,832^a^ | 30.11 | 20,057^b^ | 12.22 | 11.06 | 79 |
| 51 | Pentachlorophenol | 1,248,375^a^ | 27.67 | 30,798^b^ | 48.60 | 40.53 | 52 |
| **Other Semi-Volatile Organic Compounds (Other SVOCs)** | | | | | | |  |
| 52 | Bis 2 chloroisopropyl ether | 1,732,235^a^ | 23.50 | 872,535^b^ | 33.44 | 1.99 | 46 |
| 53 | Hexachloroethane | 8,397,293^a^ | 33.13 | 9,069,822^a^ | 26.37 | 0.93 | 32 |
| 54 | N-nitrosodi-n-propylamine | 8,261,807^a^ | 22.49 | 816,607^b^ | 34.43 | 10.12 | 52 |
| 55 | Nitrobenzene | 164,647^a^ | 23.05 | 19,659^b^ | 32.53 | 8.38 | 37 |
| 56 | Hexachlorobutadiene | 28,524,764^a^ | 26.87 | 42,109,190^b^ | 16.40 | 0.68 | 38 |
| 57 | 1,2,4-Trichlorobenzene | 14,004,112^a^ | 24.21 | 18,933,062^a^ | 24.42 | 0.74 | 30 |
| 58 | Isophorone | 110,044^a^ | 24.65 | 10,818^b^ | 22.56 | 10.17 | 56 |
| 59 | Bis 2-chloroethoxy methane | 1,108,938^a^ | 24.88 | 87,176^b^ | 25.29 | 12.72 | 32 |
| 60 | 2,6-Dinitrotoluene | 2,905,982^a^ | 27.35 | 255,382^b^ | 11.63 | 11.38 | 29 |
| 61 | 4-Chloro-di-phenyl ether | 17,414,606^a^ | 43.36 | 27,215,575^b^ | 4.81 | 0.64 | 36 |
| 62 | 2,4-Dinitrotoluene | 3,284,382^a^ | 25.02 | 224,008^b^ | 17.64 | 14.66 | 25 |
| 63 | Hexachlorobenzene | 8,516,832^a^ | 69.35 | 16,303,356^b^ | 16.32 | 0.52 | 33 |
| 64 | 4-Bromo-di-phenyl ether | 13,577,556^a^ | 62.48 | 22,868,883^b^ | 13.29 | 0.59 | 26 |
| 65 | Benzidine* | NA | NA | 5,895 | 21.9 | NA | NR |
| 66 | 3, 3 Dichlorobenzidine* | NA | NA | 2,364 | 33.4 | NA | 65 |
| % RSD-Relative Standard Deviation, DOC- Initial demonstration of capability (Section 8.2 OF U.S. EPA 625.1/2026 Method); four aliquots of reagent water spiked with the analytes of interest and analysed to establish the ability of the laboratory to generate acceptable precision and recovery, NR-Not reported in U.S. EPA 625.1/2026 Method, Mean area in each row with different superscripts (a and b) for evaluation of effect of salt addition are significantly different (P<0.05) from each other. *The results observed during the trial were inadequate due to the problematic nature of these compounds, which is evident from the very high value of MDLs, MLs, and other method acceptance criteria as reflected in EPA625.1/2016. Therefore, results from routine trials after proper optimisation of method for these compounds are reported. | | | | | | | |
